# The Hong Kong Principles for assessing researchers: Fostering research integrity

**DOI:** 10.1371/journal.pbio.3000737

**Published:** 2020-07-16

**Authors:** David Moher, Lex Bouter, Sabine Kleinert, Paul Glasziou, Mai Har Sham, Virginia Barbour, Anne-Marie Coriat, Nicole Foeger, Ulrich Dirnagl

**Affiliations:** 1 Centre for Journalology, Clinical Epidemiology Program, Ottawa Hospital Research Institute, Ottawa, Canada; 2 School of Epidemiology and Public Health, University of Ottawa, Ottawa, Canada; 3 Department of Epidemiology and Biostatistics, Amsterdam University Medical Centers, location VUmc, Amsterdam, the Netherlands; 4 Department of Philosophy, Faculty of Humanities, Vrije Universiteit, Amsterdam, the Netherlands; 5 The Lancet, London Wall Office, London, United Kingdom; 6 Institute for Evidence-Based Healthcare, Bond University, Gold Coast, Queensland, Australia; 7 School of Biomedical Sciences, LKS Faculty of Medicine, The University of Hong Kong, Pokfulam, Hong Kong SAR, China; 8 Queensland University of Technology (QUT), Brisbane, Australia; 9 Wellcome Trust, London, United Kingdom; 10 Austrian Agency for Research Integrity, Vienna, Austria; 11 Berlin Institute of Health, QUEST Center for Transforming Biomedical Research, Berlin, Germany

## Abstract

For knowledge to benefit research and society, it must be trustworthy. Trustworthy research is robust, rigorous, and transparent at all stages of design, execution, and reporting. Assessment of researchers still rarely includes considerations related to trustworthiness, rigor, and transparency. We have developed the Hong Kong Principles (HKPs) as part of the 6th World Conference on Research Integrity with a specific focus on the need to drive research improvement through ensuring that researchers are explicitly recognized and rewarded for behaviors that strengthen research integrity. We present five principles: responsible research practices; transparent reporting; open science (open research); valuing a diversity of types of research; and recognizing all contributions to research and scholarly activity. For each principle, we provide a rationale for its inclusion and provide examples where these principles are already being adopted.

## Abstract 2

Assessment of researchers still rarely includes considerations related to trustworthiness, rigor, and transparency. This Essay presents the Hong Kong Principles (HKPs), developed as part of the 6th World Conference on Research Integrity, with a specific focus on the need to drive research improvement by ensuring that researchers are explicitly recognized and rewarded for behavior that leads to trustworthy research.

## Introduction

In a quest to advance knowledge, researchers publish approximately 1.5 million journal articles each year. The assumption is that this literature can be used by other researchers, stakeholders, and the wider society because it is trustworthy, robust, rigorous, and complete [1].

The approach taken to validating research and its outcomes differs depending on the nature of the research. For example, to rigorously examine the effects of a health intervention, trial participants (human or animal) are typically required to be randomized between the intervention being studied. Many researchers advocate registration of protocols as a way to ensure transparency and to reduce bias, to discriminate between exploratory and confirmatory modes of research, and to provide insight into ongoing research projects. Subsequently, the use of reporting guidelines can help ensure complete and transparent reporting of the researchers’ methods and results. When the research is being disseminated, the research team would ensure that the associated data, materials, and any analytical code are made available as an integral part of publication. Such data sharing facilitates reanalysis of the data to check reproducibility and to perform secondary analyses.

Although some mechanisms exist to support researchers in ensuring transparency at all stages of design, execution, and reporting, there is no widespread adoption of these practices across all disciplines. There are many interwoven reasons for this. One contributing factor, we argue, is that little emphasis is placed on the rigor of research when hiring, reviewing, and promoting researchers. It seems to us more emphasis is placed on the novelty of perceived “impact” of research rather than on rigor [2]. Working together across the research sector as a whole to address this systemic issue, we believe, offers a global opportunity to improve research and impact.

**Fig 1:**
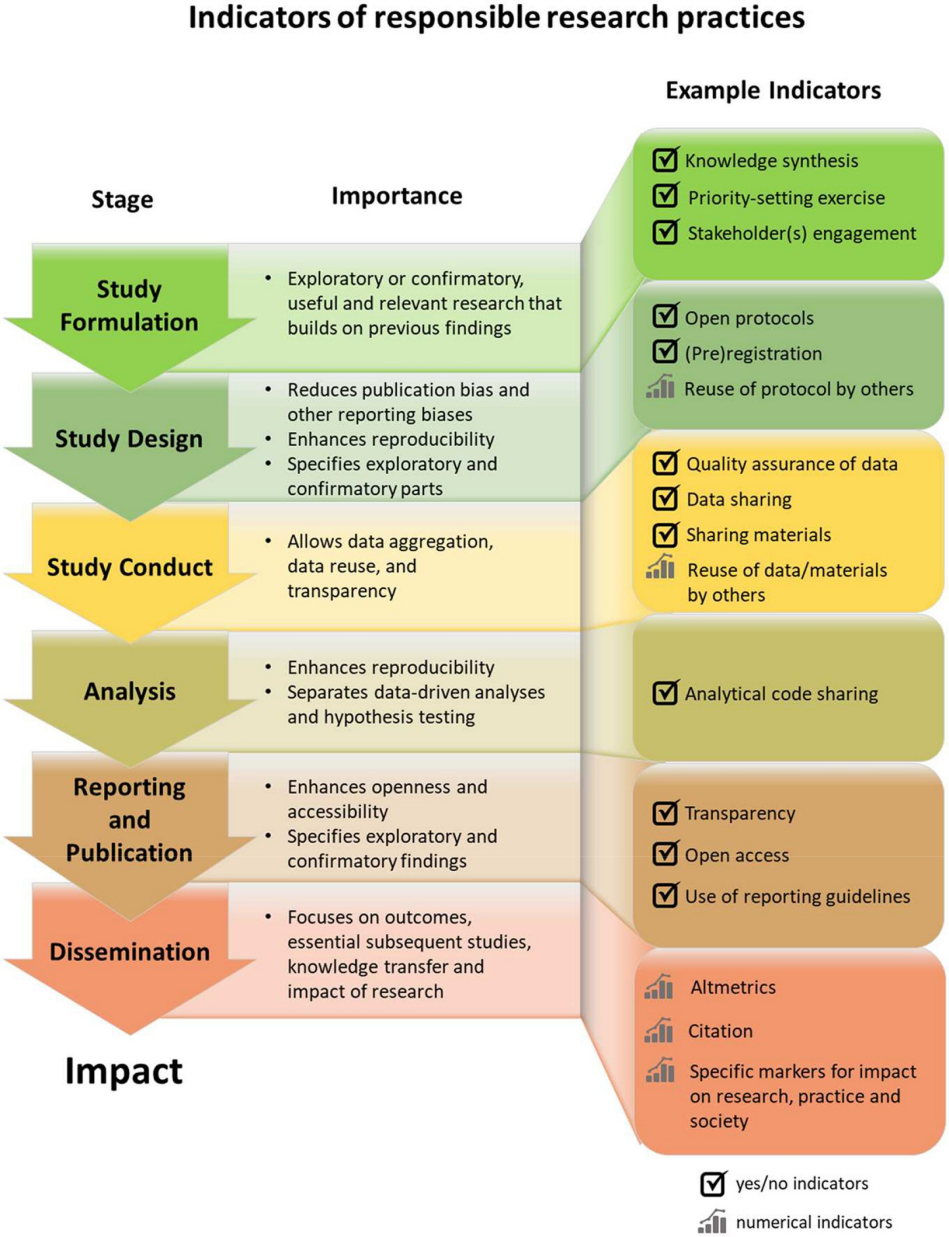
Indicators of responsible research practices.

We developed the Hong Kong Principles (HKPs) as part of the 6th World Conference on Research Integrity (WCRI) specifically to drive greater recognition for researchers who commit to robust, rigorous, and transparent practices (i.e., their careers are advanced) (see Fig 1). If implemented, the HKPs could play a critical role in evidence-based assessments of researchers and put research rigor at the heart of assessment, as well as open up research to the wider benefit of society.

We propose five principles, each with a rationale for its inclusion. The principles target exploratory and confirmatory types of research and analysis. Similarly, the principles are also applicable for quantitative and qualitative research, although there is more of a focus on assessing researchers who engage in empirical research. The principles were formulated with a focus on rewarding behaviors that strengthen research integrity that have an emphasis on responsible research practices and the avoidance of detrimental research practices [3]. We illustrate these principles with examples where we know they exist. These examples are not exhaustive, and many are relevant to more than one principle. Together, they illustrate of a breadth of approaches as to how these principles can operate at the very highest levels of international research. Early drafts of the HKPs were circulated to the 700 participants registered for the 6th WCRI. Further discussions took place during two sessions at the 6th WCRI. A penultimate version was uploaded on the 6th WCRI website after the conference. More than 100 people provided input and feedback. We acknowledge all of these valuable contributions and the global leadership of those working on the San Francisco Declaration on Research Assessment (DORA), the Leiden Manifesto, and other initiatives to promote the responsible use of metrics, which have laid the foundations for much of our work [2,4,5,6,7]. The HKPs are formulated from the perspective of the research integrity community. We, like the DORA signatories, strongly believe that current metrics may act as perverse incentives in the assessment of researchers. However, the principles outlined in this essay focus specifically on the undermining effect on research integrity [8]. We have used abbreviated versions of the wording of the HKPs below to facilitate dissemination. The complete wording of each principle is provided in Box 1.

**Box 1. Complete wording of the HKPs**

Principle 1: Assess researchers on responsible practices from conception to delivery,
including the development of the research idea, research design, methodology, execution, and effective dissemination
Principle 2: Value the accurate and transparent reporting of all research, regardless of the results
Principle 3: Value the practices of open science (open research)—such as open methods, materials, and data
Principle 4: Value a broad range of research and scholarship, such as replication, innovation, translation, synthesis, and meta-research
Principle 5: Value a range of other contributions to responsible research and scholarly activity, such as peer review for grants and publications, mentoring, outreach, and knowledge exchange

## Principles

### Principle 1: Assess responsible research practices

#### Rationale

The numbers of publications, citations, and total volume of grants are often still the dominant metrics used by research institutions for assessing and rewarding their researchers [2,4,5,6]. Providing bonuses to academics for publishing in certain journals (i.e., merit pay) is also common in many parts of the world [9–11]. These assessment criteria tell assessors little about the researchers and the rigor of their work; thus, they are not particularly “responsible” metrics, although research cited thousands of times probably indicates some measure of impact. These metrics can also be unduly influenced by field and citation practices and provide little information about a publication’s (and therefore a researcher’s) contributions to research and society. Other criteria are required to provide a broader view of markers of best practices: for example, the extent to which a researcher develops research questions with the involvement of appropriate members of the public (see Fig 1). Researchers who participate in responsible research practices, such as data sharing, which can take more time and resources, may disadvantage themselves compared to colleagues not participating in these practices. Career assessments need to acknowledge this issue.

#### Current implementation

The Canadian Institutes of Health Research’s Strategy for Patient-Oriented Research (SPOR) is a multimillion-dollar initiative to bring patients into a broad range of activities regarding research across Canadian provinces and territories [12]. Patients are now active in the development of research projects in setting priorities and formulating study questions. The Ontario response (Ontario SUPPORT Unit) has included a series of articles with patients taking a leadership role in coauthoring the content [13]. In the United Kingdom, the James Lind Alliance, funded by the UK National Institute of Health Research (NIHR), is a successful example of including patients, carers, and clinicians to develop priority-setting partnerships [14] and question formulation [15]. Other examples of citizen science across research disciplines also exist [16].

With a focus on enhancing reproducibility, the United States National Institutes of Health (NIH) have revised their application instructions and review criteria to strengthen scientific rigor and transparency [17]. One of the resources the NIH recommends is the Experimental Design Assistant (EDA) developed by the National Centre for the Replacement, Refinement & Reduction of Animals in Research (NC3Rs). This 10-module online tool was developed to assist researchers in the design and analysis of animal experiments. It includes dedicated support for randomization, blinding, and sample size calculation. It can also be used to help researchers prepare the experimental design information and analysis plan requested for grant applications [18]. The EDA is one of many tools available to help with ensuring the rigor of proposals and research more generally.

Other examples of alternative criteria include social media metrics as indicators of disseminating research [19], public lectures about the results of a research project, public engagement, and other types of events that bring together funders, researchers, and other stakeholders to work on an effective communication plan of the research program [20]. Organizations such as the Wellcome Trust are taking a holistic attitude to redefining their approach to engagement explicitly to help people feel empowered to access, use, respond to, and create health research [21].

### Principle 2: Value complete reporting

#### Rationale

Failure to publish all findings of all studies seriously distorts the evidence base for decision-making. For example, a systematic review of trials of reboxetine for treating depression found that almost three-quarters of included patients were in unpublished trials [22]; other examples across different disciplines also exist [23,24]. Selective publishing of research with positive results (i.e., publication bias) distorts science’s evidence base and has been demonstrated in a variety of disciplines including economics, psychology, and clinical and preclinical health research (e.g., [25]). Furthermore, the frequency of other reporting biases (e.g., switched primary outcomes without disclosure, and spin) is around 30% [26]. This is unacceptably high and diminishes the trustworthiness and integrity of research [11]. It also appears that promotion and tenure committees (PTCs) generally do not give sufficient importance to registering protocols and data analysis plans, full publishing of completed studies, or making data, code, and materials available [27]. These activities deserve to be credited in the assessment of researchers because they are essential for replicability, to make it possible to verify what was done, and to enable the reuse of data.

#### Current implementation

Study registration and reporting guidelines are useful tools to help improve the completeness and transparency of a very broad spectrum of research [28–31]. As part of the editorial policies of the Wellcome Trust’s open-access publishing platform (Wellcome Open Research [WOR]), authors are required to use reporting guidelines when submitting study protocols (e.g., SPIRIT) and completed studies (e.g., ARRIVE) [32]. Other funders, such as Gates Open Research [33], the NC3Rs Gateway [34], and the Association of Medical Research Charities [35], do likewise. To help reduce publication bias, WOR and other journals [36,37] use registered reports [38] (see Participating journals tab). Similarly, to promote the registration and publication of all research, the NIHR in the UK indicate that “when submitting an application to NIHR programmes for funding for a new clinical trial, the applicant must disclose past publication and trial Registration history for any relevant publications and research grants held, referenced in the application” [39]. Whereas these are examples of best practice from funders, few research institutions have incorporated them into researcher assessments [27, 40, 41].

Several research institutions (e.g., University of Toronto) are now recommending that their clinical trialists use SEPTRE [42], a web-based protocol creation and management tool. When SEPTRE is used, protocol information for trials is automatically registered in clinicaltrials.gov. This saves time and helps the researchers, and their research institutions, to maintain best publication practices (e.g., trial registration). Some journals in the social sciences, particularly psychology, use registered reports to help ensure that research is published regardless of its results [43,44].

## Principle 3: Reward the practice of open science (open research)

### Rationale

Openness (e.g., open access, open methods, open data, open code) in research is more than just access to research—it brings equality to the research process. It encompasses a range of practices across the entire life cycle of research [45]. Access to research should not be about who has the resources to pay to see behind a paywall, typically subscription journals. Healthcare and social policy decisions should be made based on access to all research knowledge rather than only a part of it [46]. A considerable amount of public funds is used for research, and its results can have profound social impact. Preclinical scientists are committing to openly share their laboratory notebooks [47] to streamline research, foster collaborations, and reduce unnecessary duplication. In an effort to deter questionable authorship practices, the Consortia Advancing Standards in Research Administration Information supports the CRediT taxonomy [48] as a way for research authors to more openly describe how each person has contributed to a research project.

Data sharing is another example of openness but is not common practice in clinical research (with some exceptions, such as genetics) [49], although patients seem supportive of sharing their data, at least of randomized trials they have participated in [50]. Data sharing is also not considered standard in many other disciplines. Without data sharing, it is difficult to check the selectivity of reports; data sharing is key to addressing concerns about reproducibility [51] and building trust [1]. There are varying estimates as to what proportion of research is made available through open-access mediums, such as open-access journals and repositories or as preprints, but it is far from 100% [52]. It seems clear that the various modalities of open science need to be rewarded in the assessment of researchers because these behaviors strongly increase transparency, which is a core principle of research integrity [45,53].

#### Current implementation

Ghent University, Belgium, has employed data sharing guidance stating, “Sound data management is a basic requirement for this [academic analysis] and provides additional guarantees for a flawless methodology, for sharing, and reusing data by other researchers in an Open Science context and for the accountability of a researchers own academic integrity" [54]. The Nanyang Technological University (NTU), Singapore, implemented an Open Access Policy in 2011. All NTU faculty and staff must deposit their final peer-reviewed manuscript of journal articles and conference papers in the Digital Repository (DR-NTU) maintained by the library upon acceptance of their publications. At NTU’s faculty of medicine, random data audits are conducted on the submitted (required) data management plans (DMPs), and checks are made to see if the final data are indeed shared on NTU’s open-access data repository DR-NTU. A coalition of funders to enforce open-access publishing in the near future [55].

To help facilitate data sharing, the University of Cambridge has introduced the concept of “data champions” [56]. Here, volunteers advise members of the research community on proper handling of research data supporting the use of the Findable, Accessible, Interoperable, and Re-usable (FAIR) research principles [57]. Delft University of Technology, the Netherlands, has taken this concept a step further and implemented it as a career assessment criterion [58]. The University of Glasgow’s academic promotion criteria explicitly allow for data sharing as a research and scholarship output (to support replication) [59].

Some journals have also established strong data sharing policies. For example, the PLOS journals “require authors to make all data underlying the findings described in their manuscript fully available without restriction at the time of publication. When specific legal or ethical requirements prohibit public sharing of a dataset, authors must indicate how researchers may obtain access to the data. Refusal to share data and related metadata and methods in accordance with this policy will be grounds for rejection” [60]. The Center for Open Science’s Transparency and Openness Promotion initiative provides information on data transparency standards for a wide variety of discipline journals [61]. Given that societal benefit is part of an emerging career assessment, clinical researchers should also respond to a growing view that patients want their data shared [50].

Open research is supported by key infrastructure compliance, such as requiring an Open Researcher and Contributor ID (ORCID) by every researcher, whereby each researcher can be uniquely identified. A recent letter from global funders committing to implementing ORCIDs for all researchers is a significant step forward [62]. This was recently implemented at the Ottawa Hospital Research Institute. In Australia and New Zealand, there is a consortium that supports ORCID nationally.

The NIH promotes the use of preprints in grant applications [63], as do all major UK public funders (e.g., Medical Research Council, UK) [64], The Wellcome Trust made them compulsory for work in health emergencies and promotes their use widely in particular for early-career researchers [65].

### Principle 4: Acknowledge a broad range of research activities

#### Rationale

A system that rewards benefit to society and encourages trustworthy and important research needs to take the different types of research into account: creating new ideas; testing them; replicating key findings; synthesis of existing research; developing and validating new tools, measures, or methods; etc. Different indicators and criteria need to be developed that are relevant to these different types and stages of research (Fig 1). This includes different timeframes of assessment for different types of research.

Incentives that encourage one fixed idea of the “right kind” of research will slow down, or even stall, progress. So-called blue-sky research that builds on chance findings or curiosity-driven research based on “out-of-the-box” thinking should be possible and encouraged as well in an academic reward system that values societal progress [66]. For example, the discovery of graphene at the University of Manchester, UK, was the result of Friday afternoon discussions outside the “normal” research activities [67]. Funders are also encouraging multidisciplinary, high-risk applications [68]. The short-term nature of academic reward cycles makes this kind of research less attractive for funders, institutions, and individual researchers. Equally, replication studies or research synthesis efforts are often not regarded as innovative enough in researcher assessments, despite their critical importance for the credibility of research or for a balanced and robust systematic presentation of all available evidence, respectively [51,69]. This is not universally appreciated by PTCs. Research on research and meta-research are practiced at, for example, METRICS (Stanford, CA, USA) [70], QUEST (Berlin, Germany) [71] (whose focus is on clinical and preclinical meta-research), the Meta-Research Center at Tilburg University [72] (Tilburg, the Netherlands) (whose focus is on the social sciences and the Open Science Collaboration), and the ongoing Psychology Science Accelerator, which consists of contributors from hundreds of universities and independent nonprofit organizations working to evaluate the barriers to replicability in psychology, in preclinical cancer biology, and across the social sciences [73]. Such activities are important to inform and improve research practices and therefore contribute to making research more reliable and relevant. The issue is that we know very little about the drivers of detrimental and responsible research practices. Furthermore, research on research (also known as meta-research) is still underfunded. As such, it is important to explicitly award this type of scholarship when assessing researchers.

#### Current implementation

Some funders have already recognized the relevance of a broad range of research activities. The Research Impact Assessment Platform (Researchfish) works to capture some of this diversity and can generate reports on the impact of a broad spectrum of funded research [74]. The Wellcome Success Framework highlights the importance of a long-term vision and shared objectives in order to take a more balanced approach to assessment [75]. The German Federal Ministry of Science and Education is funding preclinical confirmatory trials [76].

The Wellcome Trust has developed a new Longitudinal Population Studies Strategy, funded data reuse prizes [77], and supports research on research [78]. All approaches are aimed at valuing a broad range of scholarship and maximizing the value of research. The Netherlands Organization for Scientific Research is in its third call for replication studies [79]. Research on research and meta-research are also gaining momentum and now have some formal outlets. For example, *PLOS Biology* and *eLIFE* have a meta-research section in their journals [80,81]. We were unable to find any academic institution that has incorporated replication or meta-research into their career assessment portfolio [27]. NIHR requires the completion of a systematic review prior to funding any new research [82]. The NC3Rs have also promoted the importance of systematic reviews for providing a rationale for project proposals [83,84]. In the event that such a review does not exist, they provide funding to perform one.

### Principle 5: Recognize essential other tasks like peer review and mentoring

#### Rationale

As discussed alongside Principle 1, research assessments frequently focus on a narrow range of easy-to-measure metrics, including publications, citations, and funding income [2,27]. For the research ecosystem to function optimally, other research activities are also essential. Peer review remains the cornerstone of quality assessment of grants, publications, and conferences. The quality of peer-review contributions to journals and funders should also be part of assessments for promotion and tenure, as should contributions to various research infrastructure, oversight, or regulations. Equally, contributions to improvements that go beyond an individual-centered approach for assessment should be considered. These activities are currently largely missing from PTCs [27]. Contributions to developing the careers of others at all stages of their career are critical, as are contributions of various committees related to research (e.g., assuming the role of an editor). How best to do this without creating further barriers and bureaucracy, however, has long been debated [85].

Any reward system that has the whole research enterprise at heart and aims to foster a climate conducive to trustworthy and useful research with the highest regard to research integrity needs to find ways to incorporate these vital roles into its overall assessment structure. This is especially important because being a good role model as well as adequately supervising and mentoring early-career researchers are identified as top priorities in fostering research integrity [86].

#### Current implementation

Macquarie University, Sydney, Australia, has some exciting initiatives in their new academic promotion policy, which includes five pillars, one of which is in leadership and citizenship. Here, researchers can show their alignment with the university’s values and broader contribution to the university and its community [87]. Since this policy was introduced, it has been reported that the number of promotion applications increased by 50%, and the number of women promoted has also increased [88].

The University of Glasgow’s academic promotion criteria explicitly reward researchers for participation in peer review and other related activities (e.g., journal editorship) [59,89]. For this to occur, it is necessary to have organizations that can provide reviewers with a permanent identifier (a digital object identifier [DOI]) for journals that publish open reviews [90] that can be included in a researcher’s CV or that can aggregate completed peer reviews [91]. Such policies might also help promote more meaningful involvement in training in peer review [91]. The University of Exeter, UK, has developed “Exeter Academic,” a hub to help their researchers navigate career progression [92]. Leadership and citizenship are two (of five) major areas of focus. The former includes mentoring and the latter includes avenues to disseminate research knowledge from the university’s researchers.

The Finnish Advisory Board on Research Integrity (TENK) template for researcher CVs includes a broad spectrum of contributions, including mentoring and “trust in society” [93]. As a measure of mentorship, Maastricht University, the Netherlands, assesses the career progression of its PhD graduates [94]. We were unable to identify research institutions that reward researchers who have participated in training courses on high-quality mentorship [27].

The Irish Health Research Board (HRB) has a knowledge exchange and dissemination grant program providing existing HRB-funded researchers with an opportunity to seek supplementary funding for exchange and dissemination activities that can accelerate and maximize the potential translation and impact of the research findings, and learning gained, on policy or practice and health outcomes [95]. A similar scheme exists through the Canadian Institutes of Health Research [96] and the NC3Rs Skills and Knowledge Transfer grants [97] and their Crack IT open innovation platform [98].

Wellcome’s grant forms limit the number of publications applicants can submit and explicitly invite applicants to detail other achievements. This is combined with explicit guidance for panel members reminding them of the importance of taking a broad view when assessing individuals [99].

## Discussion

The HKPs focus on promoting assessment practices that strengthen research integrity by deliberately concentrating primarily on what research institutions can do to modify the criteria used by PTCs for career assessments. The emphasis on responsible research practices and the avoidance of detrimental research practices is important because these behaviors are time and resource intensive and may result in a smaller number of grants and publications. The HKPs send a clear message that behaviors that foster research integrity need to be acknowledged and rewarded. The five principles we formulated are aimed at how research institutions should incentivize, reward, and assess individual researchers for behavior that fosters research integrity within their respective organization. The HKPs do not address gender and other forms of diversity, inclusiveness, and related issues. These themes require an assessment of a group of researchers (e.g., research institution) when making decisions about funding allocations or human resources policies. Furthermore, these issues concern the social justice and societal relevance of research rather than research integrity.

### Dissemination

The WCRI Foundation [100] and the REduce research Waste And Review Diligence (REWARD) Alliance [101] will make the HKPs available on their websites. This “home” will include the principles, the signatories, infographics, translations into several languages (ongoing), future implementation plans (ongoing), and crucially, a place to highlight those who have endorsed the HKPs. Beyond journal publication, we are developing other synergistic dissemination routes.

### Endorsement and uptake

Research institutions are key to the HKPs. They are the home of current and future researchers, where promotion and tenure assessments are carried out. To help facilitate HKPs “on the ground,” local key opinion leaders and their endorsement should be included in any plan. The HKPs have been recognized by the Governing Board of the WCRI Foundation and the Steering Committee of the REWARD Alliance. We invite academic institutions, funders, other groups, and individuals to do likewise on the WCRI Foundation’s website.

We are inviting individuals and organizations to deliver brief (2–3 minutes) YouTube testimonials as to how they have implemented the HKPs (categorized by stakeholder group) and to discuss how they integrate HKPs into their, and other, initiatives. We will provide a link to these videos on the WCRI Foundation website. This approach can serve as a pragmatic way for individuals and organizations to show how they are endorsing and using the HKPs and as a nudge to others to do likewise.

To implement some of these principles is likely straightforward, although this might not be the case for all principles. To do so requires more understanding of the complexities of today’s research environment, such as the availability of institutional infrastructure, whether current CV formats are optimal to collect best practices, enabling transparency about career assessment, and considering closer alignment with policies of funders.

We would like to evaluate our approach and develop tool kits for those interested in ways to implement the five principles. We will work with signatories to take this forward. We see the HKPs as an important step along the way to improving research integrity, and we encourage an ongoing dialog to support implementation of these important principles.

## Abbreviations

DMP: data management plan
DOI: digital object identifier
DORA: Declaration on Research Assessment
DR-NTU: NTU Digital Repository
EDA: Experimental Design Assistant
FAIR: Findable, Accessible, Interoperable, and Re-usable
HKPs: Hong Kong Principles
HRB: Health Research Board
NC3Rs: National Centre for the Replacement, Refinement & Reduction of Animals in Research
NIH: National Institutes of Health
NIHR: National Institute of Health Research
NTU: Nanyang Technological University
ORCID: Open Researcher and Contributor ID
PTC: promotion and tenure committee
REWARD: REduce research Waste And Review Diligence
SPOR: Strategy for Patient-Oriented Research
WCRI: World Conference on Research Integrity
WOR: Wellcome Open Research

## References

[1] Funk C, Hefferon M, Kennedy B, Johnson C. Pew Research Centre. Trust and Mistrust in Americans' Views of Scientific Experts [Internet]. Available from: https://www.pewresearch.org/science/2019/08/02/trust-and-mistrust-in-americans-views-of-scientific-experts/. [cited 2020 Mar 25]

[2] Moher D, Naudet F, Cristea IA, Miedema F, Ioannidis JPA, Goodman SN. Assessing scientists for hiring, promotion, and tenure. PLoS Biol. 2018 3;16[3]:e2004089 10.1371/journal.pbio.2004089 29596415 PMC5892914

[3] National Academies of Sciences Engineering and Medicine. Fostering Integrity in Research. Washington, DC: The National Academies Press; 2017. 29341557

[4] American Society for Cell Biology. DORA. Declaration on Research Assessment [Internet]. Available from: http://www.ascb.org/dora/. [cited 2020 Mar 25]

[5] Hicks D, Wouters P, Waltman L, de RS, Rafols I. Bibliometrics: The Leiden Manifesto for research metrics. Nature. 2015 4 23;520[7548]:429–31. 10.1038/520429a 25903611

[6] Kretser A, Murphy D, Bertuzzi S, Abraham T, Allison DB, Boor KJ, et al Scientific Integrity Principles and Best Practices: Recommendations from a Scientific Integrity Consortium. Sci Eng Ethics. 2019 4;25[2]:327–55. 10.1007/s11948-019-00094-3 30810892 PMC6450850

[7] Biagioli M, Lippman A. Gaming the Metrics. Misconduct and Manipulation in Academic Research. Cambridge, MA: MIT Press; 2020.

[8] Leung LTF, Loock CA, Courtemanche R, Courtemanche DJ. A Cross-Sectional Analysis of the BC Children's Hospital Cleft Palate Program Waitlist. Plast Surg [Oakv]. 2019 11;27[4]:311–8. 10.1177/2292550319876664 PMC6851733 31763331

[9] Zauner H, Nogoy N, Edmunds S, Zhou H, Goodman L. Editorial: We need to talk about authorship. Gigascience. 2018;7[12]:1–4. 10.1093/gigascience/giy122 PMC6283212 30277534

[10] Quan W, Chen B, Shu F. Publish or impoverish: An investigation of the monetary reward system of science in China [1999–2016]. Aslib Journal of Information Management. 2017 1 1;69[5]:486–502.

[11] Osterloh M, Frey BS. Ranking games. Eval Rev. 2015 2;39[1]:102–29. 10.1177/0193841X14524957 25092865

[12] Canadian Institutes of Health Research. Strategy for Patient-Oriented Research [Internet]. Available from: https://cihr-irsc.gc.ca/e/41204.html. [cited 2020 Mar 25]

[13] Engaging with patients on research [Full Supplement]. CMAJ. 2018;190[Suppl.7]. 10.1503/cmaj.180816 PMC6472458 30404839

[14] The James Lind Alliance [Internet]. Available from: http://www.jla.nihr.ac.uk/. [cited 2020 Mar 25]

[15] Boote J, Dalgleish M, Freeman J, Jones Z, Miles M, Rodgers H. But is it a question worth asking? A reflective case study describing how public involvement can lead to researchers' ideas being abandoned. Health Expect. 2012;17[3]:440–51. 10.1111/j.1369-7625.2012.00771.x 22646745 PMC5060724

[16] Shirk JL, Ballard HL, Wilderman CC, Phillips T, Wiggins A, Jordan R, et al Public Participation in Scientific Research: a Framework for Deliberate Design. Ecology Society. 2012;17[2].

[17] Enhancing Reproducibility through Rigor and Transparency [Internet]. Available from: https://grants.nih.gov/policy/reproducibility/index.htm. [cited 2020 Mar 25]

[18] NC3Rs. The Experimental Design Assistant—EDA [Internet]. Available from: https://www.nc3rs.org.uk/experimental-design-assistant-eda. [cited 2020 Apr 3]

[19] Roberts Lab—School of Aquatic and Fishery Sciences. University of Washington [Internet]. Available from: http://faculty.washington.edu/sr320. [cited 2020 Apr 3]

[20] Cabrera, D. Mayo Clinic includes Social Media Scholarship Activities in Academic Advancement [Internet]. Available from: https://socialmedia.mayoclinic.org/2016/05/25/mayo-clinic-includes-social-media-scholarship-activities-in-academic-advancement/. [cited 2020 Mar 25]

[21] Wellcome's approach to engaging the public is going to change [Internet]. Available from: https://wellcome.ac.uk/news/wellcomes-approach-engaging-public-going-change. [cited 2020 Mar 25]

[22] Eyding D, Lelgemann M, Grouven U, Harter M, Kromp M, Kaiser T, et al Reboxetine for acute treatment of major depression: systematic review and meta-analysis of published and unpublished placebo and selective serotonin reuptake inhibitor controlled trials. BMJ. 2010 10 12;341:c4737 10.1136/bmj.c4737 20940209 PMC2954275

[23] Franco A, Malhotra N, Simonovits G. Publication bias in the social sciences: Unlocking the file drawer. Science. 2014;345[6203]:1502–5. 10.1126/science.1255484 25170047

[24] O'Boyle E, Banks G, Gonzalez-Mulé E. The Chrysalis Effect: How Ugly Initial Results Metamorphosize Into Beautiful Articles. Journal of Management 2020;43[2]:376–99.

[25] Chan AW, Song F, Vickers A, Jefferson T, Dickersin K, Gotzsche PC, et al Increasing value and reducing waste: addressing inaccessible research. Lancet. 2014 1 18;383[9913]:257–66. 10.1016/S0140-6736(13)62296-5 24411650 PMC4533904

[26] Dwan K, Gamble C, Williamson PR, Kirkham JJ. Systematic review of the empirical evidence of study publication bias and outcome reporting bias—an updated review. PLoS ONE. 2013;8[7]:e66844 10.1371/journal.pone.0066844 23861749 PMC3702538

[27] Rice, DB, Faffoul, H, Ioannidis, JPA, Moher, D. Academic criteria for promotion and tenure in faculties of biomedical sciences: a cross-sectional analysis of 146 universities [Internet]. Available from: 10.1101/802850. [cited 2020 Mar 25] PMC7315647 32586791

[28] Cobo E, Cortes J, Ribera JM, Cardellach F, Selva-O'Callaghan A, Kostov B, et al Effect of using reporting guidelines during peer review on quality of final manuscripts submitted to a biomedical journal: masked randomised trial. BMJ. 2011 11 22;343:d6783 10.1136/bmj.d6783 22108262 PMC3222149

[29] Turner L, Shamseer L, Altman DG, Weeks L, Peters J, Kober T, et al Consolidated standards of reporting trials [CONSORT] and the completeness of reporting of randomised controlled trials [RCTs] published in medical journals. Cochrane Database Syst Rev. 2012 11 14;11:MR000030 10.1002/14651858.MR000030.pub2 23152285 PMC7386818

[30] Tunis AS, McInnes MD, Hanna R, Esmail K. Association of study quality with completeness of reporting: have completeness of reporting and quality of systematic reviews and meta-analyses in major radiology journals changed since publication of the PRISMA statement? Radiology. 2013 11;269[2]:413–26. 10.1148/radiol.13130273 23824992

[31] Korevaar DA, Wang J, van Enst WA, Leeflang MM, Hooft L, Smidt N, et al Reporting diagnostic accuracy studies: some improvements after 10 years of STARD. Radiology. 2015 3;274[3]:781–9. 10.1148/radiol.14141160 25350641

[32] Wellcome Open Research. Policies [Internet]. Available from: https://wellcomeopenresearch.org/about/policies. [cited 2020 Mar 25]

[33] Rapid & Transparent Publishing [Internet]. Available from: https://gatesopenresearch.org/. [cited 2020 Mar 25]

[34] Maximising the 3Rs impact of NC3Rs-funded research [Internet]. Available from: https://f1000research.com/nc3rs. [cited 2020 Mar 25]

[35] Rapid & Transparent Publishing [Internet]. Available from: https://amrcopenresearch.org. [cited 2020 Mar 25]

[36] Wellcome Open Research. Preparing a Registered Report [Internet]. Available from: https://wellcomeopenresearch.org/for-authors/article-guidelines/registered-reports. [cited 2020 Apr 3]

[37] Center for Open Science. What funders are doing to support transparent and reproducible research [Internet]. Available from: https://cos.io/top-funders/. [cited 2020 Apr 3]

[38] Center for Open Science. Registered Reports [Internet]. Available from: https://cos.io/rr/. [cited 2020 Apr 26]

[39] NIHR policy on clinical trial registration and disclosure of results [Internet]. Available from: https://www.nihr.ac.uk/about-us/documents/NIHR-Policy-on-Clinical-Trial-Registration-and-Disclosure-of-Results.pdf. National Institute for Health Research. [cited 2020 Mar 25]

[40] Strech D, Weissgerber T, Dirnagl U. Improving the trustworthiness, usefulness, and ethics of biomedical research through an innovative and comprehensive institutional initiative. PLoS Biol. 2020 2;18[2]:e3000576 10.1371/journal.pbio.3000576 32045410 PMC7012388

[41] Open Science at Universities [Internet]. Available from: https://osf.io/kgnva/wiki/Universities/. [cited 2020 Mar 25]

[42] Welcome to the SPIRIT Statement website [Internet]. Available from: https://www.spirit-statement.org/. [cited 2020 Mar 25]

[43] Wicherts JM, Veldkamp CL, Augusteijn HE, Bakker M, van Aert RC, van Assen MA. Degrees of Freedom in Planning, Running, Analyzing, and Reporting Psychological Studies: A Checklist to Avoid p-Hacking. Front Psychol. 2016;7:1832 10.3389/fpsyg.2016.01832 27933012 PMC5122713

[44] Nosek BA, Ebersole CR, DeHaven AC, Mellor DT. The preregistration revolution. Proc Natl Acad Sci USA. 2018 3 13;115[11]:2600–6. 10.1073/pnas.1708274114 29531091 PMC5856500

[45] Allen C, Mehler DMA. Open science challenges, benefits and tips in early career and beyond. PLoS Biol. 2019 5;17[5]:e3000246 10.1371/journal.pbio.3000246 31042704 PMC6513108

[46] Liberati A. An unfinished trip through uncertainties. BMJ. 2004;328[531].

[47] Welcome to Open Lab Notebooks [Internet]. Available from: https://openlabnotebooks.org/. [cited 2020 Mar 25]

[48] Brand A, Allen L, Altman M, Hlava M, Scott J. Beyond authorship: attribution, contribution, collaboration, and credit. Learned Publishing. 2015;28[2]:151–5.

[49] Naudet F, Sakarovitch C, Janiaud P, Cristea I, Fanelli D, Moher D, et al Data sharing and reanalysis of randomized controlled trials in leading biomedical journals with a full data sharing policy: survey of studies published in The BMJ and PLOS Medicine. BMJ. 2018 2 13;360:k400 10.1136/bmj.k400 29440066 PMC5809812

[50] Mello MM, Lieou V, Goodman SN. Clinical Trial Participants' Views of the Risks and Benefits of Data Sharing. N Engl J Med. 2018 6 7;378[23]:2202–11. 10.1056/NEJMsa1713258 29874542 PMC6057615

[51] Munafo M, Nosek B, Bishop D, Button K, Chambers C, Percie du Sert N, et al A manifesto for reproducible science. Nature Human Behaviour. 2017;1[1]:0021. 10.1038/s41562-016-0021 PMC7610724 33954258

[52] ASAPbio—Accelerating Science and Publication in biology [Internet]. Available from: https://asapbio.org/. [cited 2020 Mar 25]

[53] National Academies of Sciences Engineering and Medicine. Open Science by Design: Realizing a Vision for 21st Century Research. Washington, DC: The National Academies Press; 2018. 30212065

[54] Using indicators in the evaluation of research [Internet]. Available from: https://www.ugent.be/en/research/research-ugent/research-strategy/indicators.htm. [cited 2020 Mar 25]

[55] Plan S. Making full and immediate Open Access a reality [Internet]. Available from: https://www.coalition-s.org/. [cited 2020 Mar 25]

[56] Research data [Internet]. Available from: https://www.data.cam.ac.uk/intro-data-champions. [cited 2020 Mar 25]

[57] Wilkinson MD, Dumontier M, Aalbersberg IJ, Appleton G, Axton M, Baak A, et al The FAIR Guiding Principles for scientific data management and stewardship. Sci Data. 2016 3 15;3:160018 10.1038/sdata.2016.18 26978244 PMC4792175

[58] Data Champions rewards [Internet]. Available from: https://www.tudelft.nl/en/library/current-topics/research-data-management/r/support/data-champions/our-data-champions/. [cited 2020 Mar 25]

[59] Academic Promotion Criteria Research Scientist Grades 7–9 [Internet]. Available from: https://www.gla.ac.uk/media/Media_498056_smxx.pdf. [cited 2020 Mar 25]

[60] Data Availability [Internet]. Available from: https://journals.plos.org/plosone/s/data-availability. [cited 2020 Mar 25]

[61] TOP Standards [Internet]. Available from: https://www.topfactor.org/. [cited 2020 Mar 25]

[62] UK Orcid [Internet]. Available from: https://ukorcidsupport.jisc.ac.uk/2018/12/funders-sign-up-to-orcid-open-letter/. [cited 2020 Mar 25]

[63] Reporting Preprints and Other Interim Research Products [Internet]. Available from: https://grants.nih.gov/grants/guide/notice-files/not-od-17-050.html. [cited 2020 Mar 25]

[64] Preprints [Internet]. Available from: https://mrc.ukri.org/research/policies-and-guidance-for-researchers/preprints/. [cited 2020 Mar 25]

[65] A more positive culture for PhD training [Internet]. Available from: https://wellcome.ac.uk/news/more-positive-culture-phd-training. [cited 2020 Mar 25]

[66] Amon A. A case for more curiosity-driven basic research. Mol Biol Cell. 2015 11 1;26[21]:3690–1. 10.1091/mbc.E15-06-0430 26515972 PMC4626053

[67] Graphene [Internet]. Available from: https://www.graphene.manchester.ac.uk/learn/discovery-of-graphene/. [cited 2020 Mar 25]

[68] New Frontiers in Research Fund [Internet]. Available from: https://www.sshrc-crsh.gc.ca/funding-financement/nfrf-fnfr/index-eng.aspx. [cited 2020 Mar 25]

[69] Camerer CF, Dreber A, Holzmeister F, Ho TH, Huber J, Johannesson M, et al Evaluating the replicability of social science experiments in Nature and Science between 2010 and 2015. Nat Hum Behav. 2018 9;2[9]:637–44. 10.1038/s41562-018-0399-z 31346273

[70] Why Meta-Research Matters [Internet]. Available from: https://metrics.stanford.edu/. [cited 2020 Mar 25]

[71] Quest [Internet]. Available from: https://www.bihealth.org/en/quest-center/mission-approaches/. [cited 2020 Mar 25]

[72] Meta Research Center [Internet]. Available from: https://metaresearch.nl. [cited 2020 Mar 25]

[73] Open Science Literature [Internet]. Available from: https://osf.io/kgnva/wiki/Open%20Science%20Literature/. [cited 2020 Mar 25]

[74] Intelligent technology to track research and evidence impact [Internet]. Available from: https://www.researchfish.net/. [cited 2020 Mar 25]

[75] How we've defined what success looks like for Wellcome's work [Internet]. Available from: https://wellcome.ac.uk/news/how-weve-defined-what-success-looks-wellcomes-work. [cited 2020 Mar 25]

[76] Bert B, Heinl C, Chmielewska J, Schwarz F, Grune B, Hensel A, et al Refining animal research: The Animal Study Registry. PLoS Biol. 2019 10;17[10]:e3000463 10.1371/journal.pbio.3000463 31613875 PMC6793840

[77] New data re-use prizes help unlock the value of research [Internet]. Available from: https://wellcome.ac.uk/news/new-data-re-use-prizes-help-unlock-value-research. [cited 2020 Mar 25]

[78] Find grants awarded [Internet]. Available from: https://wellcome.ac.uk/funding/people-and-projects/grants-awarded?scheme_id = 3569. [cited 2020 Mar 25]

[79] Replication Studies [Internet]. Available from: https://bit.ly/2H1PIt3. [cited 2020 Mar 25]

[80] Meta-Research: Evaluation and Scientometrics [Internet] Available from: https://collections.plos.org/meta-research-evaluation-and-scientometrics. [cited 2020 Mar 25]

[81] Meta-Research: A Collection of Articles [Internet]. Available from: https://elifesciences.org/collections/8d233d47/meta-research-a-collection-of-articles. [cited 2020 Mar 25]

[82] NIHR policy on clinical trial registration and disclosure of results [Internet]. Available from: https://www.nihr.ac.uk/about-us/documents/NIHR-Policy-on-Clinical-Trial-Registration-and-Disclosure-of-Results.pdf. [cited 2020 Mar 25]

[83] Funding scheme priority areas [Internet]. Available from: https://www.nc3rs.org.uk/funding-scheme-priority-areas. [cited 2020 Mar 25]

[84] The CAMARADES/ NC3Rs Systematic Review Facility [SyRF] [Internet]. Available from: https://www.nc3rs.org.uk/camaradesnc3rs-systematic-review-facility-syrf. [cited 2020 Mar 25]

[85] Credit for Peer Review: What is it Worth? [Internet]. Available from: https://scholarlykitchen.sspnet.org/2018/10/18/credit-for-peer-review-what-exactly-does-that-mean/. [cited 2020 Mar 25]

[86] Haven T, Tijdink J, Pasman H, Widdershoven G, ter Riet G, Bouter L. Do research misbehaviours differ between disciplinary fields? A mixed methods study among academic researchers in Amsterdam. Res Integrity Peer Rev. 2019;4[25]. 10.1186/s41073-019-0081-7 PMC6886174 31819806

[87] Macquarie University. New Academic Promotion scheme [Internet]. Available from: https://www.mq.edu.au/thisweek/2017/04/13/new-academic-promotion-scheme/#.XnvhhYhKg2x. [cited 2020 Mar 25]

[88] Macquarie University. Towards inclusive academic promotion. https://figshare.com/articles/EPHEA_Wollongong_Hughes_pptx/12331517. [cited 2020 May 21]

[89] Boyer, EL. Scholarship reconsidered: Priorities of the professoriate [Internet]. Available from: https://www.umces.edu/sites/default/files/al/pdfs/BoyerScholarshipReconsidered.pdf. [cited 2020 Mar 25]

[90] Reviewer Guidelines [Internet]. Available from: https://f1000research.com/for-referees/guidelines. [cited 2020 Mar 25]

[91] Track more of your research impact [Internet]. Available from: https://publons.com/about/home. [cited 2020 Mar 25]

[92] Exeter Academic. Your development [Internet]. Available from: http://www.exeter.ac.uk/staff/exeteracademic/yourdevelopment/. [cited 2020 Mar 25]

[93] A template for a researcher's curriculum vitae [Internet]. Available from: https://www.tenk.fi/sites/tenk.fi/files/CV_english_270613.pdf. [cited 2020 Mar 26]

[94] de Goede, M, Belder, R, and de Jonge, J. Academic careers in the Netherlands [Internet]. Available from: https://www.rathenau.nl/sites/default/files/2018-05/Facts_and_Figures_Academic_Careers_01.pdf. [cited 2020 Mar 26]

[95] Platforms, programmes and projects [Internet]. Available from: https://www.hrb.ie/funding/funding-awarded/platforms-programmes-and-projects/. [cited 2020 Mar 26]

[96] Population and public health. IPPH funding [Internet]. Available from: https://cihr-irsc.gc.ca/e/46949.html. [cited 2020 Mar 26]

[97] Skills and Knowledge Transfer grants [Internet]. Available from: https://www.nc3rs.org.uk/skills-and-knowledge-transfer-grants. [cited 2020 Mar 26]

[98] The CRACK IT innovation platform [Internet]. Available from: https://nc3rs.org.uk/crackit/. [cited 2020 Mar 26]

[99] Induction pack for committee members [Internet]. Available from: https://wellcome.ac.uk/sites/default/files/induction-pack-for-committee-members-2018.pdf. [cited 2020 Mar 26]

[100] The World Conferences on Research Integrity [Internet]. Available from: https://www.wcrif.org/. [cited 2020 Mar 26]

[101] The Reward Alliance [Internet] Available from: http://rewardalliance.net/. [cited 2020 Mar 26]

